# Liminality between direct and family-mediated contact in the communication of genetic information to at-risk relatives

**DOI:** 10.1038/s41431-024-01605-y

**Published:** 2024-04-16

**Authors:** Álvaro Mendes, Ainsley J. Newson

**Affiliations:** 1 CGPP – Centre for Predictive and Preventive Genetics, IBMC – Institute for Molecular and Cell Biology, Universidade do Porto, Porto, Portugal; 2 i3S – Instituto de Investigação e Inovação em Saúde, Universidade do Porto, Porto, Portugal; 3 Sydney Health Ethics, Sydney School of Public Health, Faculty of Medicine and Health, The University of Sydney, Sydney, NSW Australia

Ever since genetic test results have been able to be reported, questions have arisen regarding their implications for genetic relatives. Alongside the proband in whom the initial diagnosis is made, family members often also have an interest in the information. Being informed allows an at-risk relative to consider genetic counseling and testing, and to act in advance to prevent or mitigate future morbidity. Indeed, supporting patients to communicate risk information to their relatives is now considered as a key aspect for maximizing the benefits of genomic medicine.

However, it is also widely recognized that genetic counseling and testing among patients’ at-risk relatives remains sub-optimal [1]. This represents a critical missed opportunity to improve health outcomes. The issue is especially relevant in the context of hereditary cancer syndromes, as there is a range of effective prevention strategies and treatments that may avert an otherwise high mortality rate.

Despite this clear rationale for a familial interest in genetic (and now genomic) information, its communication and dissemination within families also remains subject to a range of ethical, legal, and psycho-social considerations. How should this information be communicated? When? By whom? To whom? Is communicating this information a duty, and if so, on whom does this fall? Should we respect a relative’s potential preference *not* to know this information? How should this be respected in the context of a busy health system and increasing mainstreaming of genomic testing [2], especially when this lies in tension with accepted norms and practices of clinical genetics?

One important aspect of debates over the family communication of genetic results is the mode of transmission within families. Two approaches have tended to be asserted in the literature: direct contact and family-mediated contact [3]. In direct contact, health professionals take the lead in reaching out to family members. In family-mediated contact, which tends to be the default practice, the proband takes the main responsibility in informing their relatives of their genetic risk. While we know that patients understand the importance of this type of communication, challenges remain. Many patients would appreciate support for family communication from healthcare professionals, and direct contact is one way this could be provided. However, direct contact remains a contentious option. We also know little about how direct contact is experienced by at-risk relatives.

The study by Öfverholm et al in this issue provides nuanced and important insights to inform ongoing debates over direct contact [4]. Direct contact is underpinned by the rationale that everyone at risk should have the opportunity to decide whether to be tested. At scale, informing relatives of a genetic risk that can be mitigated may reduce both mortality and health system burdens. Arguably direct contact also supports broader public health values, such as increasing equity in access to health-benefiting tests and enabling solidaristic actions within populations. Further reasons in support of direct contact are that a duty or responsibility to contact at-risk relatives should not fall entirely on patients and that the health system (and health professionals within it) have the knowledge, skills, and resources to ensure appropriately tailored information reaches the right people.

Knowing the experiences of at-risk relatives with receiving a letter directly from a health professional sheds light on the perspectives of an important stakeholder in this communication chain. Öfverholm et al’s findings suggest that direct contact is acceptable, but also that it should not necessarily replace family-mediated disclosure. The authors note that direct contact needs “to be implemented in a framework of ethical considerations and good practice and tailored for both the individual patient and relatives” [4]. Further, the obligation to disclose information is still felt by patients – genetic risk information is important to “hold and handle” for “oneself and others” [4].

As a result, direct contact requires a cautious approach. While its efficiency and potential public benefit provide justification for its use, direct contact can also cause harm. There are concerns that an unsolicited contact by healthcare services about a health threat may generate anxiety in relatives. Öfverholm et al. show that this is especially true for family members who were not informed about this contact by the index patient prior to receiving their letters [4].

In our enthusiasm for direct contact, we should also be careful to ensure that messaging and framing are appropriate to the information being conveyed. If a variant is well characterized and accepted as clinically actionable, then the messaging could be more directive or emphatic than if a variant is, for example, less well characterized in diverse populations. Direct communication should take care not to promote an illusion of control, a point that would perhaps be endorsed by the participants in Öfverholm et al’s study, who reflected “on the fundamental uncertainty of life” [4].

As such, it is unlikely that a single standard approach to direct contact will be suitable for all families. When designing direct contact processes, it will remain important to recognize that patients and relatives will not always feel about, or react to, the information in the same way. Prior knowledge, resourcing, and the availability of follow-up care will also impact relatives’ experiences and reactions. A liminal model of family contact and communication may be preferable. Under this approach, the option for direct contact would complement existing important relational practices within families. On this view, when family-mediated contact with relatives proves difficult —or, more rarely, when a patient actively refuses to inform relatives— healthcare professionals should first seek to understand why. It is important for them to actively inquire about the patient’s understanding of family disclosure, as there may be valid reasons behind a wish to withhold information from relatives. Additionally, it is important to consider potential communication challenges early on during pre-test counseling [5]. We must ensure that direct contact meets the preferences, values, and support needs of both patients and families.

Implementing direct contact may also require attention to non-directiveness. While debates on non-directiveness have adapted and changed to account for developments in the field, it remains a tenet of genetic counseling. On the one hand, strict respect for (a superficial, inappropriate form of) non-directiveness may inhibit a considered recommendation of direct contact. Yet it is also important to avoid a family letter being interpreted as a form of structural directivity, which could weaken autonomy.

It is very easy to overlook these various nuances in our haste to implement and mainstream genomic medicine. The necessary framework of ethical considerations and family tailoring that will remain necessary for direct contact show, we suggest, the importance of building the inherent relationality of genomics into mainstreaming practices. Of course, direct contact is indicated because genes are familial. But we should also bring relational factors in to how we design and implement direct contact. For example, life transitions, gender, family structure, and culture are important mediating factors in family communication. Follow-up visits should address possible negative reactions to direct contact. The value of familial-based care with an understanding that family communication is a nuanced process and not a one-off event should be promoted even where care is not provided by genomics professionals.

Future work in this area should encompass at least four additional dimensions. First, the question of appropriate resourcing for a high-quality model of direct contact (including facilitating initial patient-led contact, and appropriate follow-up) should be considered in light of the mainstreaming of genomic healthcare. It is crucial to equip the health system with the appropriate funding to ensure that developments in direct contact do not further strain an already over-stretched health system, which will most certainly lack the capacity to accommodate everyone to whom genetic information may be relevant. Second, non-directiveness needs further consideration. This includes both the normative question of whether non-directiveness should be upheld when questions of family communication arise, and the empirical questions of how genetic health professionals perceive their responsibilities regarding direct contact in light of non-directiveness and what they currently do about this. Third, we need to know more about how the workforce, especially non-genetics health professionals, experience direct contact. Fourth, more needs to be known regarding how patients have actually approached at-risk relatives prior to them receiving a letter from healthcare. Ultimately, we need to consider how direct contact can link with the broader health system and its evaluation.
